# Aiding Lay Decision Making Using a Cognitive Competencies Approach

**DOI:** 10.3389/fpsyg.2015.01884

**Published:** 2016-01-07

**Authors:** A. J. Maule, Simon Maule

**Affiliations:** ^1^Leeds University Business School, University of LeedsLeeds, UK; ^2^Linstock CommunicationsLondon, UK

**Keywords:** decision aids, cognitive competencies, improving everyday decision making

## Abstract

Two prescriptive approaches have evolved to aid human decision making: just in time interventions that provide support as a decision is being made; and just in case interventions that educate people about future events that they may encounter so that they are better prepared to make an informed decision when these events occur. We review research on these two approaches developed in the context of supporting everyday decisions such as choosing an apartment, a financial product or a medical procedure. We argue that the lack of an underlying prescriptive theory has limited the development and evaluation of these interventions. We draw on recent descriptive research on the cognitive competencies that underpin human decision making to suggest new ways of interpreting how and why existing decision aids may be effective and suggest a different way of evaluating their effectiveness. We also briefly outline how our approach has the potential to develop new interventions to support everyday decision making and highlight the benefits of drawing on descriptive research when developing and evaluating interventions.

## Introduction

Simon (1955) suggests that people do not make decisions in ways outlined by the rational model. He argues that the computational demands of implementing the model far exceed people's limited capacity for thinking. To overcome this problem he suggests people use simpler thinking strategies such as satisficing—choosing the first option that is reasonable rather than the one that is best. This work has provided the context for developments in both descriptive research explaining how people actually make decisions and prescriptive research outlining how we can help people make better decisions.

Although these two areas of research started from a common base, over time they have diverged. On the one hand descriptive research has increasingly focused on the thinking and reasoning processes underpinning human decision making using laboratory based methods derived from cognitive psychology and experimental economics (Weber and Johnson, 2009; Kahneman, 2011). On the other hand, prescriptive research has increasingly focused on developing methods for improving decision making drawing heavily on the rational model and management science (Watson, 1992; French et al., 2009).

This divergence has reduced the extent to which insights and developments in one area have influenced work in the other. In this article we outline how recent descriptive research on the cognitive competencies that underlie human decision making can provide a new way of developing and evaluating prescriptive approaches designed to improve human decision making. Our focus is on supporting important everyday decisions taken by members of the public such as choosing a house or apartment, a financial product, a medical procedure or whether to sue an employer for negligence following an accident at work. We do not consider support for experts or for strategic decisions taken in organizations. However, we believe that the suggestions outlined in this article have the potential to be applied in these other contexts.

We begin by reviewing prescriptive decision research, identifying the major approaches and limitations in this work. Then we briefly review descriptive decision research and consider the extent to which it can help to overcome some of the problems with prescriptive approaches. Finally, we provide suggestions about future research.

## Prescriptive research

Two prescriptive approaches have evolved to support human decision making: just in time interventions that provide support as a decision is actually being made; and just in case interventions that educate people about events that they may meet in the future so that they are better prepared to make an informed decision when these events occur.

### Just in time interventions

Initially, just in time interventions involved helping people make decisions in ways that more closely follow the rational model (Raiffa, 1968). This approach, usually called Decision Analysis, assumes that all decisions can be reduced to the same basic elements—alternatives, states of the world, outcomes, utilities and probabilities (French et al., 2009). Having derived these elements it is possible to calculate the value to the decision maker of each option. The decision maker then chooses the option with the highest value, usually expressed in terms of subjective expected utility (SEU).

Most applications of Decision Analysis support strategic decision making in organizations (Clemen and Kwit, 2001). These applications involve working with senior members of an organization over several days in facilitated sessions using a range of support techniques. These support techniques have been developed to overcome the difficulties people have when formulating problems in terms of the basic elements of Decision Analysis. We provide a brief review of two such techniques, cognitive mapping and decision trees (for a more complete review, see French et al., 2009).

**Figure 1:**
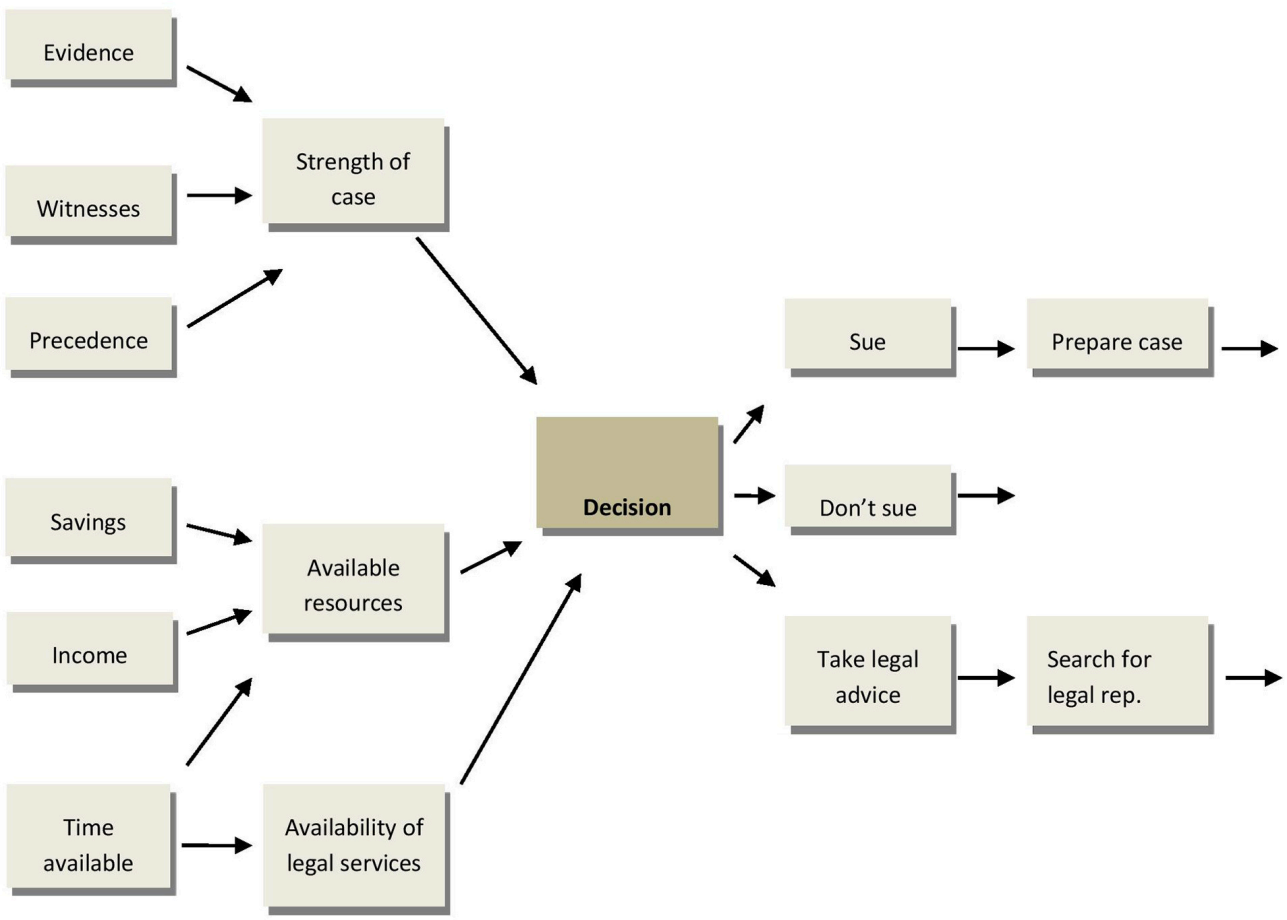
**Part of a cognitive map for a decision whether to sue an employer following an accident at work (arrows indicate casual links)**.

Cognitive mapping facilitates people's understanding of a decision problem in terms of their beliefs about the causal relationships between key factors that can affect decision outcomes. In Figure 1 we present part of a map of an everyday legal decision concerning whether, following an accident at work, a person should sue an employer and if so whether to take legal advice before doing so. The model highlights a range of legal, social and financial factors that can affect how a decision may turn out. Decision makers are prompted to take account of these factors when developing and evaluating their model of a decision problem.

**Figure 2:**
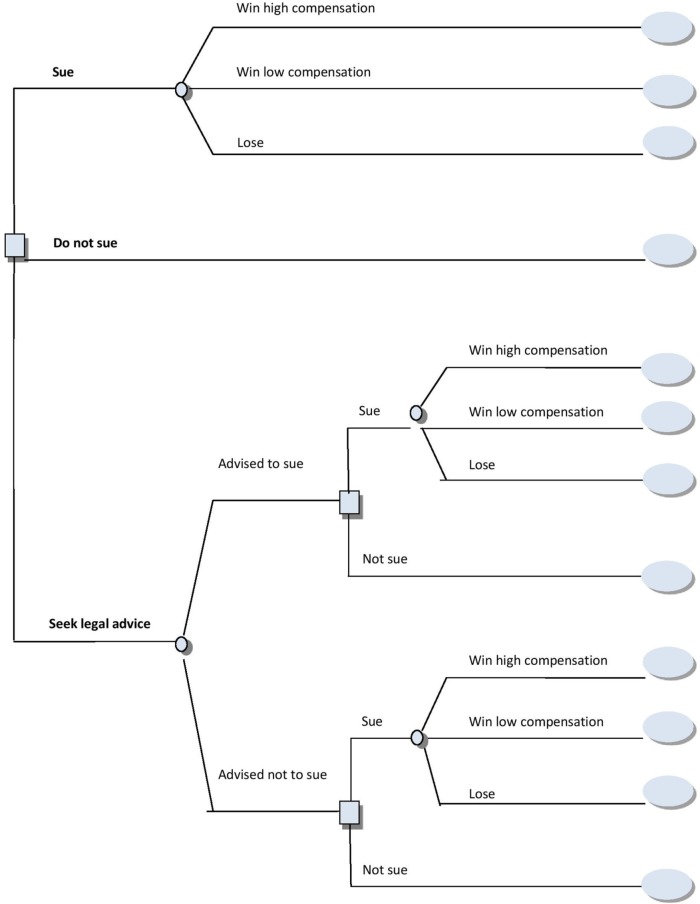
**A decision tree for a decision whether to sue an employer following an accident at work**.

Decision trees are fundamental to Decision Analysis and involve modeling the options available to a decision maker, all the possible outcomes that may occur having chosen an option, the value the decision maker places on these outcomes and the likelihoods with which each outcome may occur. This information is used to calculate the overall value of each option in terms of its SEU, thereby helping people make decisions in ways that accord with the rational model. In Figure 2 we illustrate a decision tree for the same legal problem used above to illustrate cognitive mapping. Having drawn the tree the decision maker would determine the subjective value (utility) of each outcome (represented as ellipses in the tree), the probabilities associated with each outcome (the outputs from the circles in the tree) and then calculate the SEU of each option by summing the products of each outcome utility and probability associated with this option. French et al. (2009) outline a broad range of methods for deriving the key elements of a decision tree.

Both techniques have the potential to help people develop a more elaborate representation of a decision problem. Limitations in working memory capacity make it difficult for an individual to develop, retain and evaluate a complex model of a decision problem unaided. Drawing a map or tree on paper or in a computer frees up capacity for other key cognitive activities such as assessing the adequacy of the problem representation and evaluating choice options.

Phillips (1984) suggests that a primary purpose of Decision Analysis is to develop a requisite decision model of the problem in hand, i.e., an internal representation of the problem whose form and content are sufficient for determining choice. Although this suggestion is developed in the context of groups of senior managers making strategic decisions in organizational settings, we believe it is also relevant for supporting everyday decisions. Phillips argues that models capture value judgments and their relative importance about the various advantages and disadvantages of choice options. Drawing on Harré (1976), Phillips suggests a model is a “small measure, a lesser reality ….…of the problem in hand” (pp. 33). It is simpler than reality, e.g., those aspects thought not to be important are omitted and complex relationships are often just approximated. He distinguishes between the subject and the source of a model. The subject reflects the decision maker's understanding of the problem and dictates the content of the model. The source is the rational model, leading is the rational model leading the decision maker to structure the content in terms of decision trees, cognitive maps and the like, and using such elements as acts, outcomes and consequences.

A decision maker's model of a problem evolves during Decision Analysis through group discussion and sensitivity analysis. Formally, sensitivity analysis involves changing one or more assessments either to take account of disagreements between individual group members or simply to reflect a broader range of possible values given that assessments are always approximate and uncertain. We believe that in everyday situations, sensitivity analysis can be implemented by encouraging a decision maker to think about how their choice might be affected if their current assessments were changed, e.g., a particular outcome was better or worse than their original assessment. Phillips argues that while decision makers have a sense of unease about the current model, it is not requisite so requires further development. Thus, sensitivity analysis continues until decision makers feel that no new intuitions about the problem are emerging and their sense of unease about the adequacy of the model is low. At this stage the model is said to be requisite. It is important to recognize that requisite decision models are generated, not plucked out of people's heads.

Two areas of research suggest that Decision Analysis may be relevant in everyday decision situations. First, Hodgkinson et al. (1999) show that cognitive mapping, a technique commonly used in Decision Analysis, reduces the framing bias. Since this bias is thought to occur commonly in everyday situations techniques such as cognitive mapping may have an important role in improving everyday decision making. Second, these kinds of techniques have already been applied successfully in one domain of everyday decision making concerned with health and medicine (see Bekker et al., 1999; O'Connor et al., 1999, 2009, for extensive reviews). These interventions provide evidence-based information to help patients make better health decisions. They involve presenting information about the relevant health condition; the available options; the benefits, harms and probabilities associated with each outcome; and any scientific uncertainties. This information is often presented in a decision tree (Bekker et al., 2004).

However, several problems need to be overcome if these techniques are to be used in everyday situations. First, people need to learn how to use the techniques and interpret their outputs. Hodgkinson et al. (1999) demonstrate that this may be achieved in a relatively short time period. Second, research indicates that people may be insensitive to the adequacy/completeness of their maps and trees. Fischhoff et al. (1978) showed that people are poor at assessing the completeness of a fault tree diagram associated with starting a car (fault trees are very similar to decision trees) and are insensitive to missing information. This highlights the danger of people basing their decisions on inadequate trees and maps. One possible way to resolve these two problems is to give the public pre-drawn maps and trees that experts in the area have developed and evaluated. This approach has been successful in medical/health situations (Bekker et al., 2004) though it remains untested in other everyday domains.

Third, people often find decision trees difficult because they involve generating numbers to capture utilities and probabilities. One way of resolving this is to use qualitative decision trees where people simply list the pros and cons of each outcome rather than evaluate each quantitatively.

Finally, evaluation of the effectiveness of the just in time aids remains a major problem. We discuss this issue in greater detail next.

### Effectiveness of just in time interventions

Applications of Decision Analysis in organizational settings are difficult to evaluate since they involve a unique decision, so there is no way of comparing outcomes with similar decisions not supported by Decision Analysis. Applications in health and medical settings have been evaluated in several different ways. O'Connor et al. (2009) review a large body of research showing that, in comparison to patients following standard medical procedures, those using just in time decision aids have greater knowledge about their medical condition, more accurate assessments of the probabilities of treatment outcomes and more likely to draw on their values when making the decision. In addition, patients using these aids experience less decisional conflict, feel more informed and involved in making the decision, are less likely defer making a decision and often express more satisfaction with decision outcomes. These differences are assumed to reflect better decision making. We evaluate this assumption in greater detail in a later section of this article.

Despite the difficulties outlined in this section of the article we believe that techniques such as decision trees and cognitive maps have considerable potential for supporting everyday decision making.

### Just in case interventions

This approach involves educating people about events that they may encounter in the future so that they are better prepared to make an informed decision if and when these events occur. Research has investigated this approach in the legal, financial services and medical/health domains.

#### Legal services interventions

In the legal domain, just in case interventions are designed to improve the public's knowledge about the law as a means of improving their legal decision making. For example, Mackie (2013) draws on research identifying key aspects of the law thought necessary to help people make more informed legal decisions, e.g., ability to recognize and understand the legal aspects of everyday problems and knowing where to look for relevant legal information. She designed and delivered a series of seminars to cover these key aspects. Questionnaires given to participants pre and post seminar attendance show the intervention increased understanding of legal situations and gave participants greater confidence that they understood their rights and where to seek legal advice. However, we cannot assess the long-term benefits of the intervention since its impact on later legal decision making was not investigated.

#### Financial services interventions

Research on just in case interventions in the financial services domain has occurred as a response to findings showing very low levels of financial literacy in the general public (Atkinson et al., 2006). This lack of financial literacy is assumed to be a major cause of poor financial decision making, e.g., failure to plan effectively for retirement (van Rooij et al., 2011). Interventions to improve financial literacy have included providing relevant information as inserts in pay packets, newsletters, seminars, individual consultations, education programs and information through the internet.

Reviews have concluded that evaluations taken at the end of these interventions are generally positive with participants demonstrating a better understanding of financial issues and indicating that they intend to change their behaviors in the ways advocated by the intervention. However, their impact on later financial decision making is disappointingly low. For example, Fernandes et al. (2014) showed a very small but statistically significant positive effect of financial literacy on the quality of decisions about planning for retirement, saving and avoiding high levels of debt. However, the review also showed that financial literacy levels were not determined by whether or not people had been exposed to a just in case intervention. This suggests that financial literacy is important but is unaffected by just in case interventions. De Meza et al. (2008) draw similar conclusions from their review. Taken together these findings suggest there is little evidence that just in case interventions improve financial decision making.

#### Health and medical domain

Just in case interventions in health and medical domains have involved education programs covering such issues as heart disease, diabetes, AIDS, safe sex, and avoiding the dangers of tobacco, alcohol, drugs and obesity. An important outcome from this work resonates with the findings from the legal and financial domains—simply providing relevant knowledge is rarely enough to bring about the intended changes in future decision making (Winkleby, 1994). These failures have led to a new generation of interventions that incorporate ideas from a range of social science theories concerning behavioral change. The theories drawn on mostly are the Theory of Reasoned Action, the Health Belief Model, the Theory of Planned Behavior, Social Learning Theory, Social Cognitive Theory and the Transtheoretical model—see Glanz et al. (2008) for short reviews of these theories and how they are applied in health settings. Each theory highlights key factors that need to be taken into account if an intervention is to be effective. A detailed description of the theories and how they have been applied to facilitate health interventions lies outside the scope of this article. However, next we provide some examples of the factors that these theories highlight as crucial if interventions are to succeed.

First, the Health Belief Model (Becker, 1974) highlights the importance of overcoming barriers that limit or prevent a person choosing the most appropriate option. For example, the best option may be rejected if it is perceived as unpleasant, inconvenient or time consuming to enact or in danger of revealing an unwanted outcome. Identifying and managing these barriers is crucial for developing effective just in case interventions. Second, the Health Belief Model highlights the fact that people often need a prompt indicating the need to initiate or the timeliness of the prescribed action. Without the prompt people may simply overlook the opportunity for choosing to act at all. Third, these theories highlight the importance of people believing that they have sufficient efficacy or personal control to be able to enact successfully the chosen option and receive the benefits from doing so. Finally, the Theory of Reasoned Action (Armitage and Conner, 2001) highlights the importance of behavioral intentions—an indication of an individual's readiness to perform a particular action. The strength of behavioral intentions depend upon a person's own attitude toward performing the behavior and subjective norms relating to whether people held in high regard are perceived to approve or disapprove of that behavior. This highlights the need to take account of the broader social context and the influence that others have on effective decision making.

This body of work shows that simply identifying and communicating health and medical information thought relevant by experts is unlikely to be sufficient to ensure that just in case interventions decision aids are effective.

### Effectiveness of just in case interventions

A major problem underlying just in case interventions is the lack of an underlying prescriptive theory specifying what information needs to be presented and how this should be communicated. Instead interventions are usually developed ad hoc based on what domain experts consider appropriate based on their knowledge of the area. As indicated above, to be effective these interventions have had to move beyond this atheoretical approach by drawing on theory and research on the social psychology of behavioral change. Recently two other bodies of theory have been drawn on to specify the information to be included in an intervention and how it should be presented. First, the mental models approach (Morgan et al., 2002) highlights the crucial role of user's own knowledge and understanding and the importance of specifying what is needed to supplement this and correct any errors. This approach involves comparing the mental models of the situation held by experts and the lay public, determining the kinds of decisions that people actually have to make and then identifying the knowledge gaps that are in most need of filling or correcting. Research indicates that the mental models approach is effective and has the potential to improve decisions taken months after the intervention has been completed (Downs et al., 2004).

Second, most just in case approaches are developed on the assumption that the primary driver of poor decision making is a lack of appropriate knowledge; these approaches present the additional information thought needed. This overlooks key psychological factors that determine whether and how knowledge is used when people are actually making a decision. These factors have been discussed in some detail in descriptive research. For example, people exposed to a just in case intervention may learn that it is prudent to save for retirement or to give up smoking but this may not be effective at the point of choice due to such psychological factors as a high need for immediate rather than delayed gratification (Frederick et al., 2002); or the way that options are framed at the point of choice (Edwards et al., 2001). Identifying and managing these psychological factors is crucial to the success of just in case approaches. This suggests that just in case interventions may need to draw on prescriptive insights from just in time approaches that indicate how newly acquired information is processed at the point of choice.

## Evaluation

The lack of a universal prescriptive theory underpinning just in time and just in case approaches makes evaluation of interventions problematic. Most just in time interventions use the rational model of decision making as their underlying prescriptive theory. From this viewpoint an intervention is effective if it leads people to choose the option that maximizes SEU. Although the rational model specifies very precisely the terms and calculations needed to determine SEU it does not specify the method for deriving these. As indicated earlier, just in time interventions have used a broad range of techniques for deriving key terms. However, it is not possible to directly evaluate the efficacy of these techniques since there is no way of establishing the “true” probabilities and utilities against which comparisons can be made.

These difficulties have led to a range of other evaluation methods—see Ubel (2013) for a review of these in health settings. One method involves assessing whether those exposed to the intervention know more about the decision situation and the alternatives that are on offer. The assessment usually takes place immediately after the intervention has been completed. Greater knowledge is assumed to lead to more effective decision making. Ubel is critical of this measure because it ignores whether and, if so, how people use this extra information at the point of choice. For example, forgetting may lead to crucial differences between what people recall at the end of an intervention and what is available in the future when they actually take a decision. In addition, we indicated in the previous section that this approach overlooks the possibility that the primary reason people are making sub-optimal decisions is not a lack of information but due instead to how this information is processed.

A further problem with evaluations based on user's knowledge of the situation is revealed by research showing that more information can change people's perceptions and intuitive feelings of how knowledgeable they are (Alba and Hutchinson, 2000). Hadar et al. (2013) show that giving people complex rather than simple information about investment options increases how much they know, i.e., their objective knowledge, but may lead them to feel that the situation is much more complicated than they initially thought, i.e., decrease their subjective knowledge. Decreases in subjective knowledge can have a detrimental effect by reducing a person's propensity to make a decision at all in uncertain situations given their lack of confidence about their knowledge and understanding of that situation. Conversely, if the presented information is too simple people tend to underestimate the complexity of the situation so increasing their subjective knowledge which, in turn, may lead to a lack of caution and a tendency to act when it is not appropriate to do so. These findings challenge the appropriateness of simply assuming that increases in objective knowledge lead to better decision making.

A second evaluation method uses reductions in decision conflict as a measure of intervention success. Ubel argues that this overlooks the fact that decisional conflict is often functional and induces search for additional information. For example, people presented with only the possible positive outcomes of a decision option may show little conflict when choosing that option yet be making a poor decision given they remain ignorant of possible negative outcomes.

The two measures described above are the ones used most often in health related areas, though Ubel (2013) outlines several other possible measures based on showing that those exposed to the intervention are happier with their decisions or take longer to make the decision.

Yates et al. (2003) take a rather different approach by asking people to identify the defining characteristics of a good decision. Their findings show that people take account of a broader range of factors than those currently used to evaluate interventions. For example, people indicate that good decisions occur when the outcome is better than expected; the outcome is better than would have occurred if another option had been chosen; is less costly in terms of time, money, cognitive effort; the process used overcomes difficulties experienced on previous occasions. These factors have yet to be used for evaluating just in time or just in case interventions.

Evaluation remains a major problem for both just in time and just in case interventions. In later sections of this article we propose an alternative approach based on recent developments in descriptive decision research.

## Descriptive research

From a descriptive standpoint, Simon's suggestions have provided the foundation for what is now a large body of research on the thinking and reasoning that underpins human judgment and decision making. One important feature of this research is the distinction between different types of thinking. Although the precise nature of these different types is contested (see Evans, 2008, 2010) in this article we distinguish two types: Type 1 which is broadly quick, intuitive, requires little mental effort to implement and is often based on affect, i.e., how options make people feel; and Type 2 which is analytical, deliberative, operates under conscious control and requires a good deal of mental effort to implement.

A very broad range of different forms of Type 1 thinking, often called heuristics, have been identified (Gilovich et al., 2002). These heuristics are functional in that they make complex problems tractable and allow people to resolve problems rather than becoming paralyzed by information overload. However, heuristics have the potential for error and bias though this is certainly not the case in all situations (Gigerenzer and Brighton, 2011). Errors and biases are classified in two ways. Failures of coherence occur when people violate a basic normative principle such as expressing probabilities of all possible outcomes that sum to more or less than one, or reversing their preference for options following trivial changes in the ways that options are described. Failures of correspondence occur when people make factual errors such as assuming that traveling by plane is riskier than traveling by car when, in reality, the opposite is the case.

This body of descriptive research has given rise to two areas of prescriptive research designed to improve everyday decision making. We critically review these two areas and then outline a third area that we believe has more potential for supporting everyday decision making.

### De-biasing

Arkes (1991) and Larrick (2004) review research on debiasing human judgment and decision making developed by identifying and then correcting faulty thinking. Arkes (1991) classifies known errors and biases into three categories and then suggests interventions that address distinctive aspects of each. First, strategy based errors involve the use of sub-optimal strategies, e.g., satisficing rather than maximizing expected utility. He suggests debiasing by increasing task involvement, i.e., making the outcomes more personally relevant, or by asking decision makers to justify their choices. Larrick (2004) points out that this approach can only be effective if people actually know the optimal strategy for the task in hand in the first place. In everyday decision making this is unlikely to be the case.

Second, association based errors are underpinned by Type 1 thinking such as semantic memory priming. These errors occur when initially presented/accessed information biases the information focused on later in a judgment or decision process. This leads people to over-rely on narrow/biased samples of evidence (Larrick, 2004). For example, people who are first asked to imagine a particular outcome of an event later tend to judge this outcome as more likely to occur than those not asked to imagine the outcome (Gregory et al., 1982). Imagining primes outcome related memories making these more available and memories linked to alternative outcomes less available, i.e., increasing the availability bias (Tversky and Kahneman, 1973).

Arkes argues that debiasing association errors is tricky—simply offering incentives for good performance is ineffective. Similarly, telling people about the bias and not to succumb to it is also ineffective (Fischhoff, 1975). A more promising approach involves structuring a decision maker's thinking in ways that counteract association errors. For example, “consider the opposite thinking” involves asking people to engage in counterfactual thinking—thinking about the reasons why initial judgments might be wrong (Mussweiler et al., 2000). In the example above this might involve asking people to imagine a broad range of possible outcomes before judging the likelihood that a particular one will actually occur.

Third, psychophysically based errors are associated with the Prospect Theory value function describing how people evaluate decision outcomes (Kahneman and Tversky, 1984). One example of a psychophysically based error, the framing bias, occurs when people reverse their preferences for options depending upon whether information about these options is presented in terms of gains or losses. This means that choices are dependent on how information is presented rather than people's underlying preferences. Arkes argues that debiasing can be achieved by getting people to engage in more elaborated thinking prior to choice, e.g., framing the problems in terms of both gains and losses. Although there is some support for this view (Hodgkinson et al., 1999) overall the evidence is equivocal (LeBoeuf and Shafir, 2003). These contradictory findings are due in part to researchers confusing two different aspects of framing concerning how information is presented and how that information is then represented by the decision maker (Maule and Villejoubert, 2007).

Overall, there is some evidence that debiasing may be an effective way of overcoming known errors and biases. However, there are three reasons why this approach may be limited for supporting everyday decision making.

First, the evidence is largely derived from laboratory studies. There is a paucity of research investigating the effectiveness of these techniques outside the laboratory so their impact on everyday decision making remains uncertain.

Second, debiasing techniques are designed to overcome bias associated with the use of specific heuristics, e.g., “consider the opposite thinking” designed to overcome association based errors. In everyday decisions it is often difficult to determine which, if any heuristic, is underpinning decision making. What is needed is a system for classifying and analyzing common everyday decision problems in terms of the heuristics people are using. This would allow us to predict where particular debiasing procedures are likely to be effective. A similar point is made by Larrick (2004). Without this it is difficult to know which technique people should use and how it should be evaluated.

Third, Larrick (2004) argues that many biases such as escalation of commitment are determined by many factors making it less likely that they are amenable to one specific debiasing technique

### Nudging effective decision making

Nudge is the term used to describe a relatively new way of improving everyday decision making (Thaler and Sunstein, 2008). Although nudging is similar to debiasing in that it draws heavily on descriptive research, the underlying approach is very different. Thaler and Sunstein (2008) suggest there are situations where there is general agreement about the “desired” or socially acceptable decision to take in a situation, e.g., choosing healthy eating options, to exercise frequently or to save for old age.

Nudging involves applying descriptive research to structure decision situations in ways that make it more likely that people choose the socially desirable option while still leaving them free to choose whatever they wish. Thaler and Sunstein (2008) argue that, in general, the decision context, or what they call the cognitive architecture, can be structured in ways that induce a particular mode of thinking; this leads people to choose the socially desirable option. For example, research on the default effect shows that if people are given a particular option (option A) and then given the possibility of either sticking with this option or switching to an alternative (option B) they have a strong tendency to stick with what they already have, i.e., the default option A. This preference for option A is much stronger than occurs when they are given a straight choice between the two options.

The default effect is currently being used in the UK to nudge everyday decisions concerning pensions. UK employees who do not have a pension are being automatically opted into a new work-based pension scheme, though they can opt out if they want to. Currently the average opt in rate to the pension is 92%—much higher than would be expected if employees were given a straight choice between opting in and opting out^1^.

Dolan et al. (2012) describe a broad range of nudging techniques derived from theory and research in cognitive and social psychology. These techniques have been shown to be effective in influencing people to take the socially desirable option in everyday decisions in such domains as health, finance and the environment (Thaler and Sunstein, 2008; Thaler, 2015).

Despite the success of nudging techniques there are important limitations for helping people make better decisions in the kinds of everyday situations we are interested in. First, nudging depends on being able to designate one option as socially desirable for everyone. Even where nudging is thought to be effective there are dissenting voices challenging the idea that it is possible to identify a single option as socially desirable for everyone (Guala and Mittone, 2015). This is even more problematic for the everyday decision situations we are interested where the optimal choice is likely to be different for different individuals given differences in their values. Second, the technique has been criticized for being overly paternalistic—persuading people to choose what others have designated as the optimal choice rather than allowing people freedom to choose in ways that accord with their own beliefs and values.

### Cognitive competency and decision making

An interesting new development in descriptive research focuses on key differences between people in terms of the cognitive competencies and skills underpinning their decision making, investigating how these impact on decision outcomes. From this standpoint competence concerns “the decision-making skills people need to improve their real-world decisions and to obtain better life decision outcomes” (Bruine de Bruin et al., 2012a, pp. 329).

In this article we are interested in cognitive competencies associated with components of human memory. Previous research has shown that memory processes play an important role in human judgment and decision making (Dougherty and Hunter, 2003; Reyna et al., 2003; Parker and Fischhoff, 2005; Del Missier et al., 2010, 2012, 2013). One important stream of this work has investigated the extent to which individual differences in memory related cognitive competencies can explain differences between people in terms of their propensity to fall foul of a series of well-established biases commonly revealed when people use heuristic forms of thinking. Three sets of memory related cognitive competencies have been identified that highlight differences between people in terms of: complex processing ability associated with working memory; knowledge associated with semantic memory; and experience associated with episodic memory.

Del Missier et al. (2013) argue that some cognitive biases are revealed in judgment and decision making tasks that make heavy demands on working memory, making it likely that the propensity to fall foul of these biases is linked to complex processing abilities associated with this memory component. Other biases occur in tasks that make demands on knowledge stored in semantic memory or experience stored in episodic memory making it likely that the propensity to reveal these biases is linked to differences in competencies associated with knowledge and experience. There are six areas of this work that are relevant to the current article.

First, research has shown that individuals with better working memory and executive control processes are better at resisting the framing bias, i.e., not allowing trivial changes in the wording of decision options to change preferences for these options (Del Missier et al., 2013). This finding is consistent with the view that the framing bias occurs when people are over-reliant on a simple frame derived from the surface structure and valence of the problem (Kahneman and Frederick, 2007; Maule and Villejoubert, 2007). Developing a more complex frame that overcomes the bias requires complex processing in working memory; so people who have higher levels of processing competency are better able to develop a more complex frame so resist the framing bias.

Second, complex processing abilities associated with working memory are also positively associated with the ability to implement decision rules successfully (Del Missier et al., 2010, 2012, 2013). This finding suggests that individuals who are better at complex processing are more able to undertake the computations, comparisons and aggregations that underpin the implementation of a decision rule. Successful implementation is important if decision makers are to gain the adaptive advantage that comes from using the optimal rule for the situation in hand (Payne et al., 1993; Gigerenzer and Brighton, 2011).

Third, research shows that working memory related processing competence is positively associated with the calibration of human judgment (Del Missier et al., 2013). Calibration is concerned with the extent to which people have the correct degree of confidence in their judgments, e.g., when they say they are 75% sure their judgment is correct they should actually be correct 75% of the time. Research shows a general tendency for people to be overconfident (Lichtenstein et al., 1982). Overconfidence is a problem since it increases people's tendency to choose inappropriate risky options (McGraw et al., 2004). The link between complex processing competency and confidence is consistent with an explanation that assumes people who reveal better calibrated judgments are more able to engage in cognitively demanding strategies that take account of disconfirming evidence relating to their initial judgment, rather than being dependent on less demanding strategy that focuses on confirming evidence alone (Koriat et al., 1980).

Fourth, research on the sunk cost effect shows that people continue their commitment to failing courses of action beyond the point they should (Arkes and Blumer, 1985). This effect is due in part to them being unable to ignore the resources already committed and not wanting to waste these by choosing a different action. This contradicts a canon of rational decision making—that sunk costs should be ignored, focusing instead on future costs and benefits. There is evidence to suggest that those that fall foul of this bias tend to be less competent in terms of their knowledge in semantic memory. Fennema and Perkins (2008) show that individuals who had previously taken finance, accounting and management courses highlighting the need to ignore sunk costs were less likely to fall foul of this bias. A related and somewhat surprising finding is that when differences in semantic memory competence are assessed using general measures such as letter fluency, general knowledge and vocabulary instead of actual knowledge about ignoring sunk costs, those demonstrating higher levels of competency still show reduced sunk cost effects (Del Missier et al., 2013).

Fifth, consistency in risk perception is related to semantic memory competency. Findings show that an individual's ability to generate judgments that conform to the basic principles of probability, e.g., the sum of the probabilities of all possible outcomes is 1, is related to semantic memory competence. Similar to the research on sunk cost effects, the evidence for this effect comes from research using proxy measures, i.e., letter fluency, general knowledge and vocabulary rather than direct measures of the knowledge about probability stored in semantic memory (Del Missier et al., 2013).

Finally, research indicates that semantic memory competency assessed in terms of the amount of domain knowledge people have about an area can reduce the anchoring bias. In a series of studies Smith et al. (2013) report that those with greater domain knowledge show smaller anchoring effects. In one study they showed that a group given domain relevant information later showed less anchoring than a control group presented with no information. It is noteworthy that this procedure is similar to that used in just in case interventions and may provide a blueprint for assessing the effectiveness of these intervention in the future.

These six areas show that decision effectiveness is influenced by cognitive competencies with those individuals revealing specific competencies being less likely to fall foul of particular biases. Further support for these findings comes from studies investigating the effects of age on cognitive competencies. This work builds on research showing that as people grow older they show increases in crystallized intelligence, i.e., experience and accumulated knowledge, but decreases in fluid intelligence, i.e., the ability to generate, manipulate and transform information (Salthouse, 2004). These differences have led researchers to predict that older people will reveal biases associated with complex processing to a greater extent since working memory efficiency is strongly linked to fluid intelligence. On the other hand, they should reveal biases associated with knowledge in semantic memory to a lesser extent since this memory component is strongly linked to crystallized intelligence.

These predictions are broadly supported by research. For example, older people are more susceptible to framing effects and have greater difficulty implementing decision rules, but less likely to succumb to sunk cost effects (Bruine de Bruin et al., 2012b). Although some studies fail to show these predicted effects (see for example, Mayhorn et al., 2002), Li et al. (2013) explain these anomalies in terms of a complimentary capabilities hypothesis arguing that higher levels of crystallized intelligence can offset reductions in fluid intelligence. In addition, Li et al. (2013) highlight the value of using external memory aids to alleviate processing loads for older adults. We believe that this is also likely to be true for younger adults when faced with complicated everyday decisions that require complex processing in working memory. This will be particularly the case for those low in complex processing competency.

The implications of these findings for developing and evaluating just in time and just in case interventions are discussed next.

#### Implications for just in time interventions

Existing just in time decision aids are effective in part because they support complex processing underpinning working memory and executive control thereby increasing a decision maker's competency to deal with this aspect of decision making. Interventions such as cognitive mapping and decision trees allow decision makers to develop and maintain their representation of a decision problem externally on paper or in computer software rather than having to hold it in working memory. This frees up working memory capacity for other important activities such as appraising and modifying the problem representation and evaluating options to determine which to choose. These interventions also reduce the processing demands by providing guides about the sequence of activities needed to make a decision rather than leaving decision makers to determine this for themselves.

There are untested predictions from this line of reasoning. First, just in time interventions that support complex working memory processing such as cognitive mapping and decision trees are likely to be effective in everyday situations where framing and decision rule implementation pose difficulties for decision makers. However, their impact should be less where framing and decision rule implementation are relatively straightforward and/or where the major cause of poor decision making involves the lack of appropriate knowledge. In the latter situations interventions linked to semantic memory competencies should be more effective, e.g., just in case interventions. Second, just in time interventions are likely to be particularly effective for those individuals low in complex processing competencies, including older people, given these interventions support this processing.

#### Implications for just in case interventions

Just in case interventions are effective in part because they deliver important decision related information at one moment in time that is stored in semantic memory so that it is available later when a relevant decision situation arises. From this standpoint, just in case decision aids can be thought to increase semantic memory competence by providing the knowledge needed to make an effective decision.

There are untested predictions from this line of reasoning. First, just in case interventions should be effective in situations where sunk costs, inconsistency in risk perception and anchoring pose particular difficulties for decision makers since these biases can be reduced by increased knowledge. However, just in case interventions are unlikely to be effective in situations where, at the moment of choice, there are heavy processing demands from such activities as framing and decision rule implementation. This view is consistent with suggestions by De Meza et al. (2008); they suggest that a major limitation on the effectiveness of just in case interventions is that they overlook the thinking and reasoning processes involved at the moment of choice.

Second, just in case interventions are likely to be less effective when individuals already have relevant knowledge and experience; this includes older people since they have had longer to acquire this knowledge. Third, just in case interventions will be more effective when the information presented is determined and delivered in ways that increase the likelihood that it will be incorporated and retained in semantic memory. Earlier we argued that the mental models approach is designed in this way. By building on users' existing knowledge, supplementing gaps and correcting misapprehensions key knowledge presented in an intervention is better integrated in semantic memory so more likely to be available later at the point of choice.

## Applying competence research in prescriptive settings

Having shown the importance of cognitive competencies for our understanding of just in time and just in case interventions, in this section we outline two examples of how research on one aspect of research, concerned with complex processing in working memory, may be applied to improve existing interventions.

### Supporting meta-cognitive process in learning environments

A primary aim of research on meta-cognitive processes in learning environments is to find ways of increasing understanding of a problem and its solution by presenting different external representations of the problem and support for managing and integrating these representations internally (Renkl et al., 2013). Limitations in working memory capacity make integration difficult so researchers have developed procedures designed to provide external support. For example, Schwonke et al. (2009) investigated ways of integrating probability equations and a tree diagram (very similar to a decision tree) for understanding and solving probability problems. They show that simply presenting different representations is not effective because people have difficulty integrating them. Renkl et al. (2013) review different ways to support complex processing within working memory in order to facilitate integration. Two of these are particularly relevant for this article.

First, self-explanation prompts ask individuals to explain solution procedures to themselves while working through a problem. This form of prompting builds on the self-explanation effect—those that actively engage in explaining solution procedures to themselves achieve better learning outcomes (Chi et al., 1989). Berthold et al. (2009) argue that these prompts facilitate the integration of different representations of a problem and in doing so increase understanding of that problem. However, they show that this only occurs in relatively simple situations; in complex situations the processing demands of these kinds of prompts exceed capacity limits so reduces rather than increases understanding. They overcome this problem by simplifying the activities involved in self-explanations, e.g., asking users to fill in blanks of statements about the relationship between different representations of the problem rather than prompting with open questions.

We believe that a similar procedure can help people develop a requisite decision model when using decision trees to support everyday decisions. A key element in developing a requisite model is sensitivity testing where people vary their current assessments of the probabilities and values associated with particular outcomes. Simplified prompts have the potential to support the complex processing necessary when generating and integrating the outcomes of sensitivity testing. For example, simple prompts with blanks for inserting judgments about the highest and lowest possible outcome values can provide support for developing a range of different models of the problem when looking for the requisite model. In addition, Phillips (1984) argues that a key element in determining if a model is requisite or not can be linked to feelings of unease about the current model—these feelings signal the need for further modeling. Again, prompting questions about these feelings and how to interpret them can be used to guide decision makers as to when their model is requisite.

Second, goal-operator prompts are designed to help people identify and explicate key sub-goals as they are being achieved. Research shows that these prompts foster knowledge acquisition in problem solving (Aleven and Koedinger, 2002) and improve understanding of key aspects of problems (Schwonke et al., 2009). In the context of decision trees this could involve prompting decision makers to identify when a key sub-goal has been completed, e.g., all possible outcomes that may follow choice of an option have been identified. Research suggests that these prompts facilitate understanding of the problem in hand, and in doing so help in establishing a requisite decision model.

These are just two examples of how research on facilitating complex processing in working memory can be used to develop a more effective just in time intervention based on decision trees. There are likely to be many others.

### Training on increasing working memory capacity

Recent research has shown that working memory capacity can be expanded through training. Morrison and Chein (2011) identify two approaches. Strategy training teaches people strategies for increasing the amount of information that can be retained in working memory over time. It is domain specific—it focuses on a particular component such as the rehearsal loop, uses specific training procedures designed to improve the functioning of that component and then evaluates the extent to which the intervention improves the performance of that component. Core training on the other hand is domain-general and typically involves a battery of different cognitive activities. For example, COGMED (Holmes et al., 2009) involves training based on a battery of tasks such as backward digit span and visual tracking and its effectiveness is measured across a wide range of general cognitive tasks, e.g., tasks involving cognitive control, such as the Stroop test and measures of fluid intelligence (Klingberg et al., 2002). Changes in fluid intelligence are particularly important, given the link between this and the use of cognitive heuristics discussed earlier in this article. There is also evidence suggesting that training benefits can still be present 6 months after training (Holmes et al., 2009).

Core training may have the potential to facilitate just in case interventions through improved learning of decision relevant information. However, we believe that its greatest impact will be on just in time interventions. The increased capacity derived through training should lead to similar benefits to those described earlier, i.e., those individuals with greater working memory capacity will be less prone to particular biases such as the framing bias and/or better able to implement decision rules effectively.

However, some important issues about this research remain unresolved (Morrison and Chein, 2011). For example, where a battery of tasks is used it is difficult to determine which ones are effective in inducing improvements in performance. Also, research has often not ruled out the possibility that improvements are simply placebo effects associated with people being more motivated after training, rather than the training *per se*.

Despite these problems, working memory training seems to have the potential for improving everyday decision making, though research is needed to test this contention.

## Implications of competencies research for aiding decisions

The central tenet of our argument is that the competencies approach provides a different way of conceptualizing how and why decision aids lead to better everyday decision making and provides insights about how these interventions may be improved and evaluated.

From this standpoint, just in time interventions such as decision trees can be seen as enhancing a decision maker's competence to engage in complex processing underpinning working memory. We have shown that thinking about just in time aids in this way draws in other bodies of theory and research hitherto neglected by those responsible for developing these interventions. In particular, we have discussed how the use of prompts and general working memory training can be used to improve the effectiveness of just in time interventions. We believe that these are two of what may be many such modifications.

Also, this approach provides different ways of evaluating these interventions by testing the extent to which they reduce or even eliminate those biases associated with complex processing competence, e.g., the framing bias.

Similarly, just in case interventions can be seen as enhancing a decision maker's knowledge (semantic memory competence) and experience (episodic memory competence). This suggests that these aids can be enhanced by drawing on bodies of theory and research on how these different memories operate and can be enhanced. Earlier we discussed the mental models approach (Morgan et al., 2002) which builds on users' existing knowledge, supplementing gaps and correcting misapprehensions rather than simply presenting what domain experts think is necessary (the standard practice from those developing just in case interventions). This approach, which facilitates long term retention, has been shown to improve decisions taken months after an intervention is completed (Downs et al., 2004). We believe that there are likely to be other competencies associated with semantic and episodic memory that can be incorporated in to just in case interventions. For example, remembering the past, imagining the future, and engaging in mental simulation processes (Greenberg and Verfaellie, 2010; Schacter et al., 2012) are crucial to human decision making and are linked to episodic memory competency (Del Missier et al., 2015).

In addition, this approach provides a distinctive way of evaluating just in case interventions by testing the extent to which they reduce or even eliminate those biases associated with knowledge and experience, e.g., sunk cost and anchoring biases while leaving those that are not, e.g., framing bias, unaffected.

## Knowing which intervention to use

Knowing which techniques are likely to be effective in a particular decision situation is an enduring problem for those wishing to support human decision making. The competencies approach has the potential to provide some insights about how to solve this problem. Del Missier et al. (2012) discuss how different decision tasks draw on cognitive competencies to different degrees. For example, complex processing competencies may be more critical when the decision involves complex information about options, consequences and other relevant information (Dretsch and Tipples, 2008; Del Missier et al., 2010). This suggests that just in time interventions are likely to be useful in these kinds of decision situations. In contrast to this, other decisions can be resolved using simpler strategies based either on learned associations and simple heuristics (Glöckner and Witteman, 2010) or on specialist knowledge (Bruine de Bruin et al., 2012b) and recognition primed processes (Klein et al., 2010). The latter two are particularly likely to draw on knowledge and experience so are likely to be linked to competencies associated with episodic and semantic memory. Research is needed to develop a taxonomy of everyday decision tasks in terms of the demands they place on different cognitive processes and to test our predictions about which kind of intervention is likely to be most effective for improving decision making.

## Conclusion

The competencies approach provides a different way of thinking about when and why decision aids lead to better judgment and decision making. It provides a way of developing and evaluating just in time and just in case interventions based on identifying and supporting the cognitive competencies necessary to overcome known biases that occur in particular decision situations.

Although these suggestions have considerable promise there is a need for further research. For example, work is needed to investigate further the idea that interventions such as cognitive mapping and decision trees do indeed facilitate complex cognitive processing; and, if so, whether they are effective at eliminating biases linked with competencies associated with complex processing such as the framing bias, difficulties implementing decision rules and calibration but ineffective for biases linked to knowledge and experience competencies such as sunk costs and anchoring.

Similarly research is needed to test whether those just in case interventions that are successful achieve this through enhancing knowledge and experience based competencies and are effective at reducing or eliminating biases such as sunk costs and anchoring but ineffective for those associated with complex processing competencies.

Research verifying these suggestions will not only provide further support for the importance of cognitive competencies in human decision making but also help us determine better which interventions are likely to be effective in a particular everyday decision making situations and why this is the case. This will redress a lack of theory specifying how to match interventions to problems.

The competencies approach may also have the potential to help us develop new interventions based on principles related to how best to improve the functioning of those aspects of cognition that are crucial to the decision task in hand. We illustrated this earlier by showing how working memory research can be used to develop and test improvements in just in time and just in case interventions.

We have focused only on cognitive competencies. Some key competencies may be non-cognitive. For example, decision may induce very strong emotions that may need to be regulated if they are not to have a disruptive effect on the decision making process (Castellanos et al., 2006). The importance of emotions in determining decision biases has already been shown to be crucial in sustaining the sunk cost bias (Bruine de Bruin et al., 2014). Developing interventions that support emotion regulation competencies may provide a very different but equally effective approach to helping people make better everyday decisions in situations that induce strong emotions.

Finally, the work reviewed in this article highlights the need to re-establish the links between prescriptive and descriptive decision research. On the one hand we have shown how new developments in descriptive research can provide: a different way of thinking about why decision aids may be effective; a set of principles for modifying existing decision aids; and suggest better ways of evaluating the effectiveness of decision aids. On the other hand, we have shown how prescriptive research can provide an effective test bed to develop and evaluate theories drawn from prescriptive research.

### Conflict of interest statement

The authors declare that the research was conducted in the absence of any commercial or financial relationships that could be construed as a potential conflict of interest.

## Footnotes

^1^http://www.nestpensions.org.uk/schemeweb/NestWeb/includes/public/news/NEST-Corporation-and-NEST-Scheme-annual-reports-and-accounts-2014_15.html
